# Proliferative response of peripheral blood mononuclear cells in anti-Hu antibody-associated patients with paraneoplastic neurological syndrome and their depressant effect on small cell lung cancer cells

**DOI:** 10.3892/mmr.2014.2975

**Published:** 2014-11-19

**Authors:** LIANG YIN, HONGDANG QU, QIMING CHEN

**Affiliations:** Department of Neurology, The First Affiliated Hospital of Bengbu Medical College, Bengbu, Anhui 233004, P.R. China

**Keywords:** anti-Hu antibody, pathogenesis, cytoimmunity, paraneoplastic neurological syndrome, proliferation

## Abstract

Previous studies have suggested that common antigens that exist in neurons and tumor cells trigger cross immunoreaction, which attacks the neurons and tumor cells simultaneously. This action leads to paraneoplastic symptoms and slowed tumor growth. The present study co-cultured peripheral blood mononuclear cells (PBMCs) from anti-Hu antibody-associated patients with paraneoplastic neurological syndrome (PNS) and the small cell lung cancer cell line NCI-H446 (H446) *in vitro*. In the PNS group, no significant difference was observed in the proliferation index of PBMCs and H446 cells between the mixed or separate cultures. The proportion of CD4^+^ T cells in patients with PNS (76.54±3.96%) was significantly higher (P<0.05) compared with the healthy control individuals (51.75±17.3%). In conclusion, the sensitized specific T cells in the PBMCs of PNS patients predominantly comprised of CD4^+^ T cells, which had no inhibitory effect on small cell lung cancer cells *in vitro*.

## Introduction

Paraneoplastic neurological syndrome (PNS) is a neurological syndrome, which is closely associated with malignancy. Among the various types of malignant tumor that cause PNS, small cell lung cancer (SCLC) is the most common (1). Several studies have suggested that cross immunoreaction, caused by common antigens expressed in the neuron and tumor cells of patients with PNS, is directed at the tumor and the nervous system, limiting tumor growth and leading to the occurrence of PNS (2). Previous studies have indicated that inside the body of patients with PNS, numerous neuron-specific antibodies exist, including the anti-Hu antibody, and these studies demonstrated that humoral immunity has an important function in the incidence of PNS (3,4). However, a number of studies have suggested that humoral immunity is not the only pathogenic factor and that T cell-mediated cytoimmunity is also involved in the pathogenesis of PNS (5,6). The present study used the Avidin-Biotin Complex (ABC) immunohistochemical method and western blot analysis to detect neuron-specific antibodies (anti-Hu antibody) in the serum of patients with PNS. The positive antibody of peripheral blood mononuclear cells (PBMCs) and SCLC cell lines (H446) from patients with PNS were co-cultured *in vitro*. The present study aimed to observe whether PBMC was able to inhibit the growth of H446. In addition, the percentage of CD4^+^ and CD8^+^ T cells following stimulation with interleukin (IL)-2 was monitored, based on humoral and cellular immunity, to examine the pathogenesis of PNS.

## Materials and methods

### Subjects

#### PNS group

A total of seven patients with SCLC and PNS with a high titer of anti-Hu antibodies were enrolled from the Department of Neurology, The First Affiliated Hospital of Bengbu Medical College (Bengbu, China). Among these patients, five were male and two were female with an average age of 57.7±8.4 years (mean ± standard deviation). Among these patients, three had paraneoplastic sensory neuropathy two had paraneoplastic limbic lobe cephalitis and two had paraneoplastic encephalomyelitis.

#### SCLC group

A total of six SCLC patients without PNS (anti-Hu antibody negative) were included from the same hospital. Of these patients, four were male and two were female with an average age of 55.1±9.0 years (mean standard ± deviation). The present study was conducted in accordance with the Declaration of Helsinki and was conducted with approval from The Ethics Committee of Bengbu Medical College (Bengbu, China). Written informed consent was obtained from all patients.

#### Control group

A total of eight health workers from The First Affiliated Hospital of Bengbu Medical College were included, comprising five males and three females with an average age of 48.8±8.5 years (mean ± standard deviation).

All PNS patients were diagnosed using the criteria for PNS (7). The lung primary tumors removed from patients during surgery were confirmed to be SCLC following pathological examination.

### Immunohistochemical method

Frozen sections (6 μm) of a healthy brain (obtained within 6 h mortality and without infectious disease or PNS) were obtained. Following drying at room temperature for 10 min, the sections were fixed with acetone (Tianjin TBD Biotechnology Development Center, Tianjin, China) for 10 min and then blocked for 30 min in 10% calf serum albumin (Hangzhou sijiqing Biological Engineering Materials Co., Hangzhou, China) dissolved in phosphate-buffered saline (PBS; Tianjin TBD Biotechnology Development Center). The sections were incubated overnight at 4°C following the addition of several different serum dilutions and were then incubated with polyclonal goat anti-human IgG (cat no. BA-3000; Vector Laboratories, Inc., Burlingame, CA, USA) at room temperature for 30 min and with ABC (cat no. PK-4000; Vector Laboratories, Inc.) for 40 min. The reaction was terminated with PBS following staining of the slides using benzidine (Sigma, Santa Clara, CA, USA) for 5 min. Finally, the slides were dehydrated using alcohol and xylene (Fengyuan Pharmaceutical Co., Bengbu, China) and were then assessed using a conventional microscope (WJ12-50, XSB-14; Shanghai Precision Instrument Co., Shanghai, China).

### Western blot analysis

#### Neuronal nucleus extraction

Nuclei were extracted from healthy neurons via the improved Blomstrand and Hamberger method (8). The neuron extracts were abstracted via sodium dodecyl sulfate-polyacrylamide gel electrophoresis (SDS-PAGE; Fengyuan Pharmaceutical Co.) and transferred onto a nitrocellulose membrane (Shanghai Tuoran Biological Technology Co., Shanghai, China) based on the Towbin method (9). These were then fixed in 5% skimmed milk and dissolved in PBS for 60 min, following which a variety of serum dilutions [≥1:1,000; 1 g bovine serum albumin + 0.01 PBS (100 ml) + 0.08g NaN_3_] were added prior to incubation overnight at 4°C. The reaction was terminated with PBS and compared with the standard protein following incubation of the extracts with polyclonal goat anti-human IgG at room temperature for 30 min and with ABC for 40 min. The extracts were then colored using benzidine (Fengyuan Pharmaceutical Co.) for 5–8 min.

#### HuD cloning method for purification of proteins

The HuD cloning purified protein, a product of genetic engineering obtained from Professor Jean-Yves Delattre (Salpetriere Medical College of Curie University, Paris, France), was separated via SDS-PAGE. The following steps were identical to those described above in the method for the neuronal nuclei extraction.

### Culture of cells and staining with carboxyfluorescein diacetate succinimidyl ester (CFSE)

The PBMCs were separated from the venous blood via gradient centrifugation (778 g for 20 min at 20°C) and seeded in 24-well plates. CD3 monoclonal antibody (obtained from Professor Carding, Department of Medical Microbiology, University of Pennsylvania, Philadelphia, USA), at a final concentration of 5 g/ml and rhIL-2 (Changsheng Gene Pharmaceutical Co., Changchun, China), at a final concentration of 50 μg/ml, were placed in each well. The PBMCs and H446 cells were cultured in RPMI 1640 cell culture fluid (10% new-born calf serum +150,000U/l gentamicin) in 5 % CO2 saturated humidity incubator at 37°C for 5–7 days and H446 was obtained and conventionally cultured. The PBMCs and H446 cells were diluted with PBS at densities of 5×10^6^ and 3×10^6^/ml, respectively. The cell suspensions were stained using CFSE (Molecular Probes, Carlsbad, CA, USA; final concentration 1 μmol/l) at 37°C for 10 min and rinsed twice with PBS. The cell suspensions of PBMCs and H446 were detected using flow cytometry (FACS Calibur; BD Biosciences, Franklin Lakes, NJ, USA) and the initial fluorescence intensity was analyzed using Modifit software (Pc Win98/NT; Verity Software House, Inc., Topsham, ME, USA) and preserved. Followed staining with CFSE, the PBMC and H446 cells were cultured together at densities of 2×10^5^ and 5×10^4^/ml, respectively, for 3 days.

### Assessment of cell proliferation

Following culture, the cell suspensions were added to different fluorescent labeled monoclonal antibodies, namely, IgG1/IgG2, CD3/CD4 and CD3/CD8 (BD Biosciences). The CFSE fluorescence intensity was detected and compared with the initial fluorescence and the proliferation index of total PBMC, CD4^+^ and CD8^+^ T cells and H446 cells were obtained using ModFit software. The proportion of each PBMC subgroup was analyzed using CellQuest software (BD Biosciences).

### Statistical analysis

The statistical significance of differences in the proliferation index between two types of culture was assessed using a Wilcoxon matched-pair signed-rank test. Differences between three groups were assessed via Kruskal Wallis H test. For all statistical analyses, P<0.05 was considered to indicate a statistically significant difference.

## Results

### ABC

**Figure 1:**
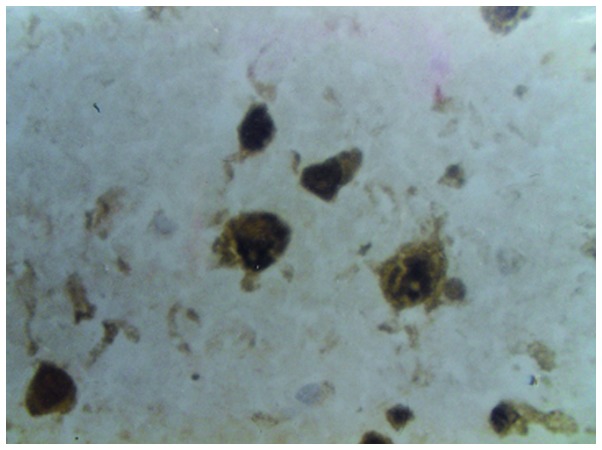
Serum of patients with PNS exhibited uniform staining of neuronal nuclei in the 6 μm frozen sections from normal brain, with negative nucleolus.

From screening a total of 20 patients with PNS and 120 patients with SCLC, staining was observed in the neuronal nuclei of the frozen sections of normal brain in 89% of the PNS patients and 40% of the SCLC patients, with negative nucleoli. No reaction was observed with glial cells in the nervous tissue or in the liver and kidney tissue. The antibody concentration in the serum of the PNS group was 1:1,000–64,000, whereas the value in the SCLC group was 1:1,000–8,000. No staining was observed in the control groups (Fig. 1).

### Neuronal nucleus extraction

**Figure 2:**
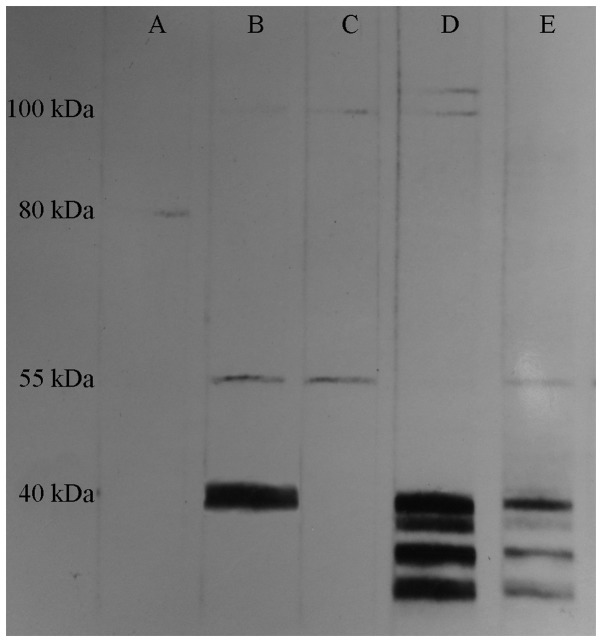
Western blot analysis revealed how the neuron-specific anti-Hu antibodies identified protein antigen with a molecular weight of 35,000–40,000 Da in the neuronal nuclei extract. (A) Negative control; (B) positive control; (C) negative sera; (D) positive sera.

The anti-Hu antibody identified mainly protein antigen with a molecular weight (MW) between 35,000 and 40,000 Da in the neuronal nuclei extract. The serum from a total of two SCLC and 16 PNS patients had the above-mentioned specific bands, whereas these bands were not observed in the control group (Fig. 2).

### Purification of proteins

**Figure 3:**
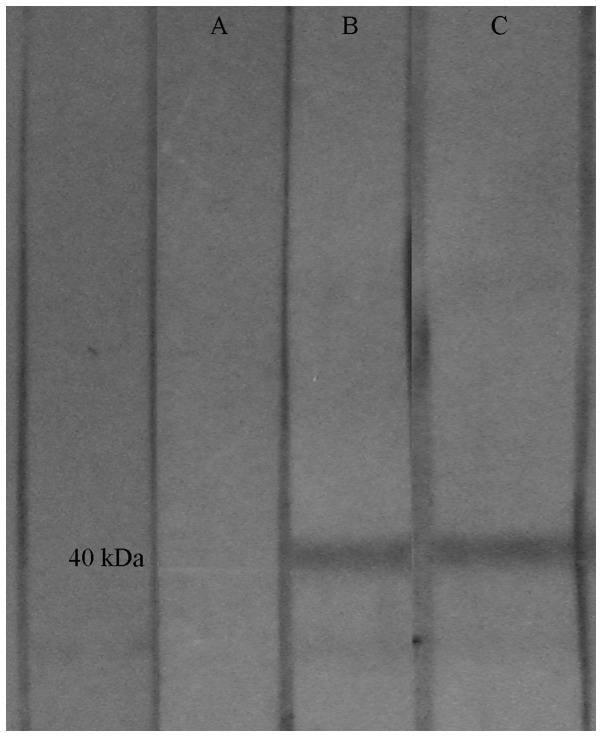
Western blot analysis revealed how the neuron-specific anti-Hu antibodies recognized HuD cloning purified protein with a molecular weight of 40,000 Da. (A) Negative control; (B) positive control; (C) positive sera.

The anti-Hu antibodies and cloned HuD purified protein formed a single band at 40,000 Da. The band observed in the PNS and SCLC patients was identical to that obtained in the neuronal nuclei extract method, whereas these bands were not observed in the control group (Fig. 3).

### Cell proliferation index

**Figure 4:**
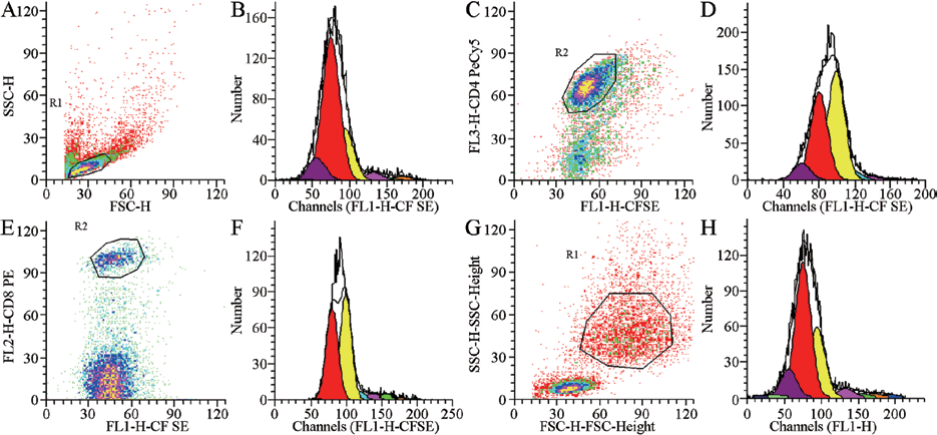
Proliferation dynamics of the cells. (A and B) Proliferation dynamics of total PBMCs. (C and D) Proliferation dynamics of CD4^+^ T cells. (E and F) Proliferation dynamics of CD8^+^ T cells. (G and H) Proliferation dynamics of NCI-H446 cells co-cultivated with PBMCs. PBMCs, peripheral blood mononuclear cells; CFSE, carboxyfluorescein diacetate succinimidyl ester.

In the PNS and SCLC groups, no significant differences were observed in the proliferation index of the H446, total PBMCs, CD4^+^ and CD8^+^ T cells between the mixed cultures and separate cultures. In the control group, the proliferation index of the total PBMCs, CD4^+^ and CD8^+^ T cells was increased significantly when the PBMC and H446 cells were cultured together compared with when they were cultured separately (P<0.05). The proliferation index of the H446 cells decreased significantly (P<0.05; Table I and Fig. 4).

### Comparison of the percentages of each subgroup

**Figure 5:**
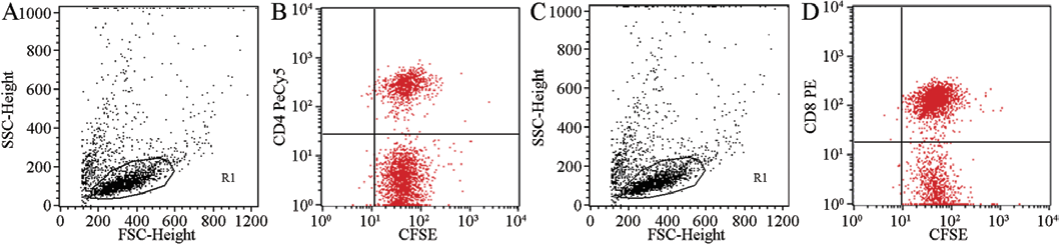
Two-dimensional lattice figure of the percentage of T cell subsets. (A and B) Two-dimensional lattice of the percentage of CD4^+^ T cells. (C and D) Two-dimensional lattice of the percentage of CD8^+^ T cells. CFSE, carboxyfluorescein diacetate succinimidyl ester.

Following separate cultivation or co-culture with H446, the proportion of CD4^+^ T cells increased significantly in the PNS group compared with the control group. The proportion of CD4^+^ and CD4^+^/CD8^+^ T cells increased significantly and the proportion of CD8^+^ T cells decreased significantly in the SCLC group (P<0.05). The proportion of CD4^+^ and CD4^+^/CD8^+^ T cells in the SCLC group increased significantly and the proportion of CD8^+^ T cells decreased significantly compared with those in the PNS group (P<0.05–0.01). No significant difference was observed between the separate culture and mixed culture in the same group (Table II and Fig. 5).

## Discussion

Cross immunoreaction triggered by a common antigen is involved in the pathogenesis of numerous autoimmune diseases (10). Previous studies have indicated that the appearance of PNS is also associated with cross immunoreaction (11,12). It has been previously demonstrated that following repeated administration of serum immunoglobulin G from patients with PNS into animals, neuromuscular junction abnormalities were observed in the electrophysiology (13). It has also been observed that particular anti-Hu antibody-positive SCLC patients have an improved status compared with antibody-negative patients (14). Therefore, it has been hypothesized that cross immunoreaction, which is directed at the tumor and the nervous system, exists in patients with PNS, thereby limiting tumor growth and resulting in the occurrence of PNS (15).

With the development of immunotechnology, the neuron-specific anti-Hu antibody, has been identified inside several patients with PNS. Several studies have demonstrated that the appearance of PNS is closely associated with the immune response of antibodies mediated in the body (16). Previous studies have revealed that subgroups of anti-Hu antibodies are widely distributed in the serum, nervous system and tumor tissue via the immunohistochemical method (17,18). The present study demonstrated that anti-Hu antibodies reacted significantly with the neuronal nuclei of the central nervous system and reacted weakly with the cytoplasm. The results of the western blot analysis indicated that anti-Hu antibodies in the serum recognized protein antigens with a relative MW between 35,000 and 40,000 Da in the neuronal extract from the human cerebral cortex. Therefore, detecting anti-Hu antibodies may be a promising method in the early diagnosis of PNS (19).

To investigate the proliferation of lymphocytes and tumor cells in an accurate manner, flow cytometry was performed to detect cell proliferation. CFSE stably combines the proteins in cells to produce fluorescence. Fluorescence intensity is reduced by half when cells divide in half. Flow cytometry detects fluorescence and cell surface molecules, which enables analysis of the proliferation kinetics of the lymphocytes subgroup (20). The results demonstrated that in a mixed culture *in vitro*, the H446 cells accelerated the proliferation of PBMCs among healthy individuals and the proliferation of PBMCs inhibited the growth of H446 cells. However, a similar condition was not observed in the PNS and SCLC patients, possibly due to the following reasons. Previous studies have demonstrated that the percentage of CD4^+^ T cells and the ratio of CD4^+^/CD8^+^ decreased in patients with PNS, which in turn led to reduced immune function; therefore, the PNS PBMC showed now clear inhibitory effects on tumor cells *in vitro* (21,22). Another study demonstrated that, in the peripheral blood of PNS patients, the percentage of CD4^+^ T cells and the ratio of CD4^+^/CD8^+^ cells is decreased, leading to a degree of immune function inhibition in patients with PNS, but no clear inhibitory effects on tumor cells cultured *in vitro*. Healthy individuals have normal immune function and cell proliferation. In the H446 culture, the immune function may respond against tumor cells and inhibit the growth of H446.

Although the proportion of CD4^+^ T cells and the ratio of CD4^+^/CD8^+^ T cells in PNS and SCLC patients decrease, clone amplification may be prioritized in this part of the cell body if sensitization of the antigen specific T lymphocytes occurs inside. The present study demonstrated that stimulating the activation of cell proliferation is prioritized due to IL-2, regardless of the culture condition. The CD4^+^ T cell ratio of PNS patients was significantly higher compared with healthy individuals following culture, subsequent to augmenting PBMCs with IL-2 for 5–7 days. In addition, the percentage of CD4^+^ T cells and the ratio of CD4^+^/CD8^+^ cells in the patients with SCLC were higher compared with those of healthy individuals. Therefore, the sensitized specific T cells in the PBMC of patients with PNS and SCLC were predominantly composed of CD4^+^ T cells. Previous studies have revealed that the immune response associated with anti-Hu antibody syndrome involves the participation of cellular and humoral immunities (23,24). Studies have found that IgGl and IgG3 activate complement, however, the reaction is weak and is confined to a small area of the nervous system. In addition, natural killer cells have not yet been found (25,26). This reaction may be a non-complement mediated cytotoxicity reaction and non-antibody dependent cell-mediated cytotoxicity (25). Anti-Hu antibodies can identify antigens, including HuD and HuC. HuD is considered to be the only antigen that is expressed in patients with SCLC (27). Previous investigation of the peripheral blood lymphocyte membrane phenotype of anti-Hu antibody syndrome patients has demonstrated that CD4^+^ T cells can directly attack the HuD antigen and are involved in cell-mediated nervous system damage and anti-tumor effects (28). In addition, based on pathological results, the number of brainstem and spinal cord neurons of patients with PNS is significantly decreased, with a large number of inflammatory lymphocytes infiltrating the blood vessels, similar to that of lymphocyte distribution in the sleeve sample. The majority of inflammatory lymphocytes are CD19^+^ B and CD4^+^ T cells (15). This previous study also demonstrated that CD4^+^ T cells are involved in cell-mediated damage of the nervous system. The results revealed that sensitized specific T cells in patients with PNS and SCLC were mainly CD4^+^ T cells in the body (15). This observation is similar to that of a previous study, showing that CD4^+^ T cells have an important function in antitumor immunity (28).

The present study demonstrated that, following culture *in vitro*, the proportion of CD4^+^ T cells and CD4^+^/CD8^+^ T cells in the SCLC group was significantly higher than that in the PNS group and the ratio of CD8^+^ T cells was decreased significantly. The specific reasons for this require further investigation.

## Figures and Tables

**Table I:** Comparison of the cell proliferation index in the control group by two culture methods (mean ± standard deviation; n=8).

Training method	H446	Total PBMCs	CD4^+^ T cell	CD8^+^ T cell
Separate culture	8.56±3.34	8.05±5.51	7.43±4.63	7.69±5.84
Mixed culture	6.97±2.52^a^	8.90±5.67^a^	8.60±4.83^a^	8.93±5.73^a^

a P<0.05, compared with the separate culture.

PBMCs, peripheral blood mononuclear cells.

**Table II:** Comparison of CD4^+^, CD8^+^ and CD4^+^/CD8^+^ ratio in the three different groups (mean ± standard deviation).

	CD4^+^ T cells (%)		CD8^+^ T cells (%)		CD4^+^/CD8^+^ ratio	
			
Group	Separate Training	Mixed Training	Separate Training	Mixed Training	Separate Training	Mixed Training
Control	56.01±18.62	51.75±17.3	37.30±21.3	36.02±18.71	2.64±2.83	2.15±1.88
PNS	82.4 ±5.12^a^	76.54±3.96^a^	14.51±3.26	17.41±7.31	5.18±2.39	4.74±2.42
SCLC	88.99±2.61^b^^c^	89.36±3.77^b^^d^	9.72±2.49^b^^c^	7.91±1.56^b^^d^	9.74±2.85^b^^c^	11.61±1.95^b^^d^

a P<0.05,

b P<0.01, compared with the control group;

c P<0.05,

d P<0.01, compared with the PNS group.

PNS, paraneoplastic neurological syndrome; SCLC, small cell lung cancer.
